# ImageParser: a tool for finite element generation from three-dimensional medical images

**DOI:** 10.1186/1475-925X-3-31

**Published:** 2004-10-01

**Authors:** HM Yin, LZ Sun, G Wang, T Yamada, J Wang, MW Vannier

**Affiliations:** 1 Center for Computer-Aided Design, The University of Iowa, Iowa City, IA 52242, USA; 2 Department of Radiology, The University of Iowa, Iowa City, IA 52242, USA; 3 Department of Civil Engineering, University of Illinois, Urbana, IL 61801, USA; 4 Department of Diagnostic Radiology, Tohoku University, Sendai 9808574, JAPAN; 5 Department of Radiology, National Taiwan University, Taipei, TAIWAN ROC; 6 Department of Radiology, The University of Chicago, Chicago, IL 60637, USA

**Keywords:** Breast imaging, image segmentation, biomechanical analysis, meshing, finite element method (FEM)

## Abstract

**Background:**

The finite element method (FEM) is a powerful mathematical tool to simulate and visualize the mechanical deformation of tissues and organs during medical examinations or interventions. It is yet a challenge to build up an FEM mesh directly from a volumetric image partially because the regions (or structures) of interest (ROIs) may be irregular and fuzzy.

**Methods:**

A software package, ImageParser, is developed to generate an FEM mesh from 3-D tomographic medical images. This software uses a semi-automatic method to detect ROIs from the context of image including neighboring tissues and organs, completes segmentation of different tissues, and meshes the organ into elements.

**Results:**

The ImageParser is shown to build up an FEM model for simulating the mechanical responses of the breast based on 3-D CT images. The breast is compressed by two plate paddles under an overall displacement as large as 20% of the initial distance between the paddles. The strain and tangential Young's modulus distributions are specified for the biomechanical analysis of breast tissues.

**Conclusion:**

The ImageParser can successfully exact the geometry of ROIs from a complex medical image and generate the FEM mesh with customer-defined segmentation information.

## Background

Diagnostic imaging devices such as CT, MRI and PET scanners are able to produce three-dimensional (3-D) descriptions of various features such as tissues and organs. In a computer, these images are some data to describe the intensity at each spatial point of a volume. The interpretation of the dataset requires special training and depends on the experience. Researchers have introduced a variety of algorithms to visualize 3-D medical images, and to extract the geometric information of objects from volumetric image data [1-3].

In recent years, the finite element method (FEM) has widely been used to simulate the mechanical deformation of tissues and organs during examinations or interventions [4-6]. To build up an FEM mesh from a medical image, the contour information of segmented regions of interest (ROIs) need to be first extracted from a volume of data [7,8]. Then, the volume is meshed into nodes and elements, and material properties are endowed to each element in accordance with the segmentation information [9]. By further applying the boundary conditions and mechanical loadings on the corresponding nodes or elements, commercial FEM software packages such as ANSYS and ABAQUS may calculate the mechanical stress and strain, and predict the deformation and motion in the field of view.

**Figure 1:**
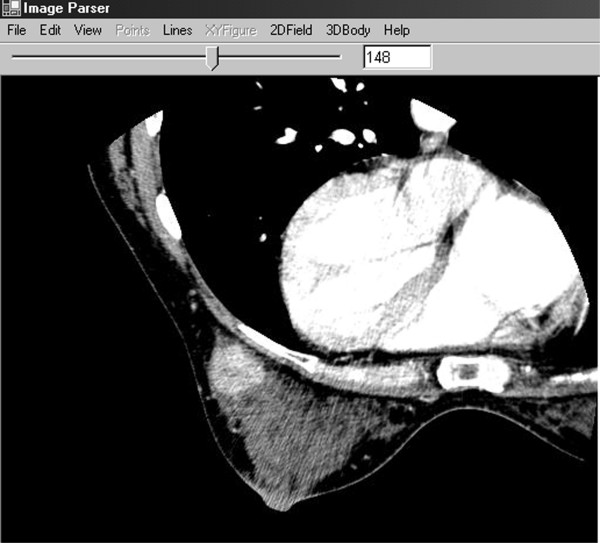
**The Interface of ImageParser When Loading a 3D Image. **The image is automatically shown slice by slice with the slice number shown in the text box, and the interval between two slices can be changed. Clicking the slide bar or text box, we can focus on the current slice; double clicking the window area, we can navigate the image slice by slice again; and dragging the slide bar or inputting the slice number in the text box, we can jump to the desired slice.

The purpose of this work is to establish an FEM model to simulate the deformation of a woman breast based on mammography compression. A patient's breast may include three kinds of tissues: fatty, parenchyma, and cancerous tissues [6]. During the examination, the breast is squeezed by two flat paddles to obtain an image with a good contrast. The dependence of the relative deformation carries information on the mechanical properties of the tumors and masses. Thus, the FEM is a powerful tool to simulate this kind of deformation. The breast need first be separated from the context of a biomedical image which includes some other organs and the different tissues of the breast are then segmented. As seen in Figure 1, parenchyma has a cloud-like shape and three tissues are fully mixed in some regions. While existing modeling techniques [e.g., [1,2]] may be applied, a significant amount of 3-D elements have to be introduced because the geometric shapes of the constituent tissues are fairly irregular with fuzzy boundaries. It is not optimal to apply those techniques to our research and clinical studies due to the requirements on the computational efficiency.

In this paper, a software package called the ImageParser is developed to generate an FEM model from 3-D medical images. While aiming at the imaging segmentation, mesh generation, and deformation simulation of heterogeneous breast tissues, the method is applicable to the many biomedical imaging and biomechanical analysis of soft/hard tissues such as mammography and cardiovascular imaging. This software uses a semi-automatic method to detect the objective constituents from the context of an image including neighboring tissues and organs. It segments an image based on customer-defined grayscale ranges, and meshes tissues into elements with a customer-defined size. Inputting the generated FEM mesh into an FEM program, we can calculate the mechanical deformation under specific boundary conditions and mechanical loadings. The ImageParser is written in Microsoft Visual C# .NET (Microsoft Development Environment 2003 Version 7.1), and can be integrated into a high-level image analysis environment with a good extendibility and scalability.

## Description

### Overview

**Figure 2:**
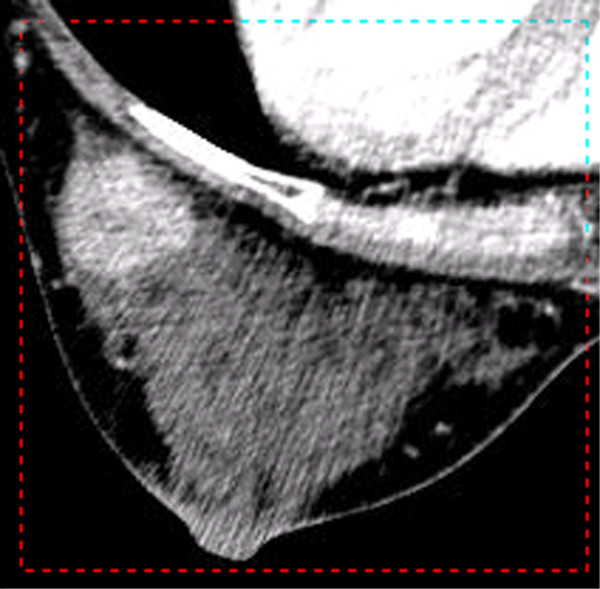
**The Region of Interest in a Slice. **Under the function of selecting ROI, press the left button of the mouse and drag the mouse. When the dashed line rectangle covers ROI, release the button. All the slices will be shown as this selection.

The ImageParser provides a window style GUI as shown in Figure 1. A 3-D image is loaded and shown slice by slice. We can focus on any slice and edit it. While the image can be displayed in the color mode, in graphics analysis, we use 8-bit grayscale to describe a voxel. The RGB color can always be transformed into grayscale according to a desirable equation [10]. The size of voxel can be user-defined. The image can then be segmented into the real organs. Here we use Figure 1 as an example to show the procedure for generating an FEM mesh using the ImageParser. In Figure 1, the breast is the selected ROI within the axial CT image including the breast, ribs, and organs in the thorax. To obtain its geometric information, we first isolate it from other unwanted regions by selecting a rectangular region as shown in Figure 2. It is noted that this selection also works for all other slices so that when we select the ROI, we need work on the most representative slice and reserve enough space not to truncate the wanted region in other slices. Because the shape of the breast is irregular, we can still see the rib and part of the thorax in the ROI as well as some regions with the background color. We need further detect the borderline of the breast in each slice.

**Figure 3:**
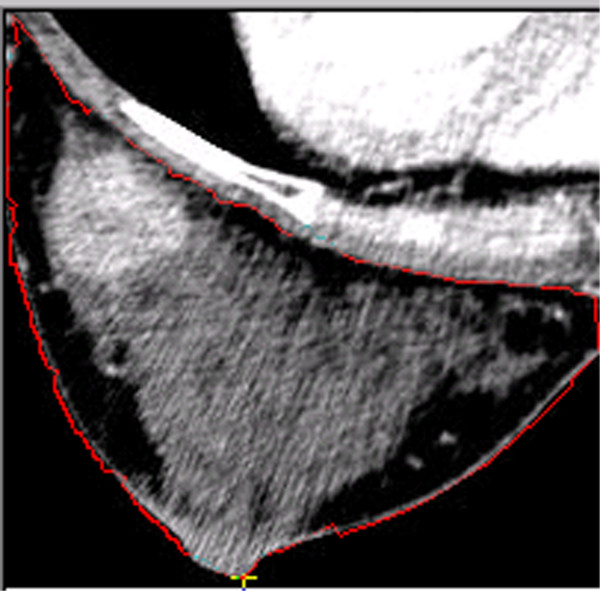
**Selecting and Detecting the Borderline of the Breast in ROI. **Under the function of selecting Outline, click the left button of the mouse on a point close to the borderline. The software will detect the closest point of the borderline and mark it with a yellow cross. After click the next point close to the borderline, the software will automatically detect the closest borderline between the previous point and this one. Repeat this procedure. The borderline is finally detected.

This software provides a semi-automatic interface to detect the borderline of the breast as shown in Figure 3. We use the computer mouse to select some key points on the borderline, and then the software will automatically detect the borderline between the key points. Because the borderline changes from one slice to another, the software is designed to automatically detect the borderline of the neighboring slice using the known borderline as a seed. Repeating this procedure, the software can detect the borderlines for all slices. The algorithmic details will be provided in the next section. Based on these borderlines, we can reconstruct the surface of the breast. It is noted that for some special cases that the borderlines are fuzzy or irregular, the software cannot effectively detect the borderlines. However, we can manually select more key points in the first slice, and process other slices similarly. Then, based on our experience we can always manage to obtain the borderlines with a high precision.

**Figure 4:**
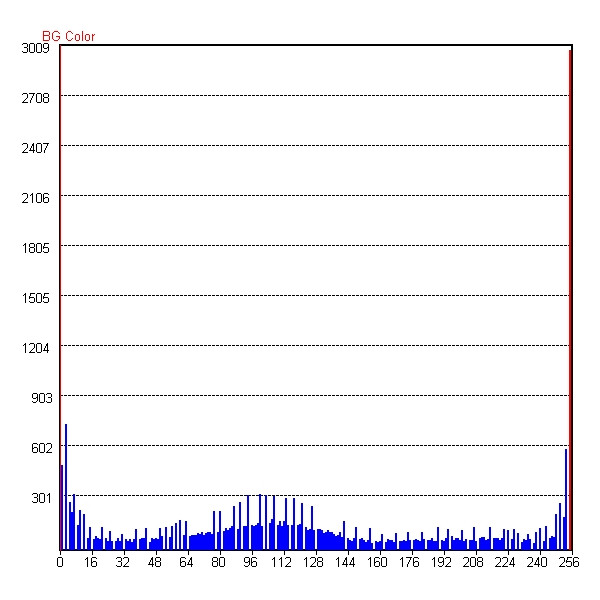
**The Grayscale Histogram of ROI. **Horizontal axis denotes a grayscale (*G*), and vertical axis a number of voxels. *G *= 0 is the background color of black; *G *= 255 the color of white. From the grayscale distribution corresponding to the tissues in Figure 3, we can define the grayscale range for fat, parenchyma, and tumor tissues.

Figure 3 shows three types of tissues in the breast: the black area representing fatty tissue, the gray area representing parenchyma, and the light area representing the tumor. Because these tissues have different mechanical properties, we need segment them out of the breast as new ROIs. Here we use the grayscale to classify the voxels. From the grayscale histogram in Figure 4, we can find the grayscale ranges corresponding to the different tissue types. With the grayscale range for each tissue, the software can map the voxels onto corresponding categories. For example, in this figure we can use the grayscale from 0 to 64 to represent the fatty tissue, 64 to 144 the parenchyma tissue, and 144–256 the cancerous tissue.

**Figure 5:**
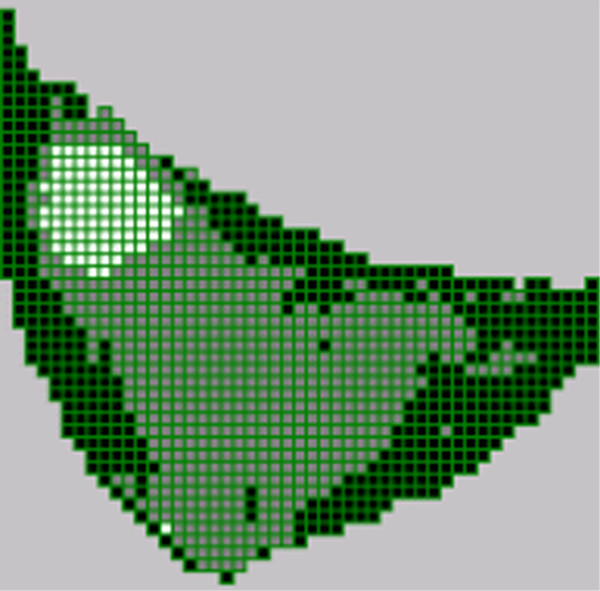
**The FEM Mesh of the Breast. **The selected region is meshed by cuboidal elements. The color of black denotes fat; gray parenchyma; and white tumor. The green lines are the boundary of elements. Though only one slice is shown here, elements are also generated for some other slices so that the 3D FEM mesh of the Breast is obtained.

While we are able to directly output the segmentation information based on the voxels, it cannot be effectively used by any FEM software since the whole breast includes more than ten million voxels. We therefore mesh the breast into larger elements based on the specific requirements of precision and computation capability. Figure 5 shows the FEM mesh of one slice with three tissues marked by different colors. Extending this procedure to all slices and considering the slice thickness and element size, we can obtain the 3-D FEM mesh of the breast. While we take cuboidal elements as an example to generate the mesh at this stage, we can also mesh the breast into other elements such as tetrahedrons.

After the mesh of the breast is generated, we can implement it into an FEM software package to simulate the mechanical deformation of the breast with given material constants of all the tissues and appropriate boundary conditions.

### Borderline Detection

A medical image typically includes many kinds of organs and tissues. However, biomedical engineers may only be interested in a small number of regions in a complex medical image. While certain algorithms have been developed to automatically detect the surface of the 3-D image [1-3,11], the object surface may not be well defined because the ROI is in the context of the complex image, and the boundary is not clear especially for some soft tissues. We have to use our knowledge to isolate ROIs from the image. Therefore, we propose to develop a semi-automatic method as described in the following steps.

1. We first focus on one slice. When the first point (*x*_0_, *y*_0_) is selected close to the borderline, a function is then used to search the most possible border point in the square region with the left-top point (*x*_0 _- *s*, *y*_0 _- *s*) and the right-bottom point (*x*_0 _+ *s*, *y*_0 _+ *s*). Here *s *is a customer-defined parameter with a default value as 3 pixels. In this region, the gradient of each point Δ(*x*, *y*) is defined by a Laplace operator [12]



so that



where *f*(*x*, *y*) denotes the intensity at (*x*, *y*). The detected point is the one with the different color from the background and with the maximum value of Δ(*x*, *y*)/[(*x *- *x*_0_)^2 ^+ (*y *- *y*_0_)^2 ^+ *ε*] where *ε *is a customer-defined parameter with a default value as 0.1 to prevent the singularity on the point (*x*_0_, *y*_0_). The detected point is denoted as (*x*_1_, *y*_1_). It is noted that the Laplacian normalized by the distance of the point to the selected seed point is to make the neighboring points have a higher priority to be detected. At certain regions, the borderline may not be clear or two borderlines are close enough, in which cases the program will not get lost.

**Figure 6:**
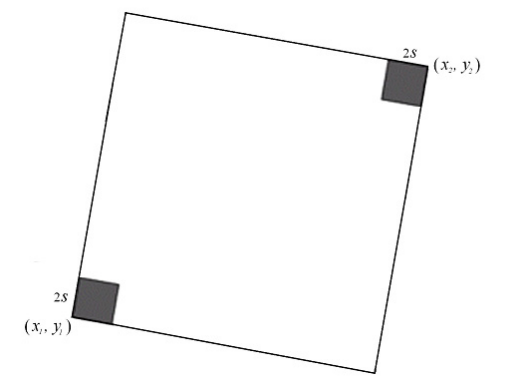
**Detecting Borderline between Two Points (*x*_1_, *y*_1_) and (*x*_2_, *y*_2_). **First find the most possible border point in two dark regions. Then, treat the new point and the left old one as same as (*x*_1_, *y*_1_) and (*x*_2_, *y*_2_), and find the next border point. Repeat this procedure until the distance between two points is less than *2s*. Connecting all points orderly, we obtain the borderline.

2. We start to select the next point of the borderline. After we manually select one point visually close to the borderline following the method of detecting (*x*_1_, *y*_1_), the software can adjust the location of the point and detect the second point (*x*_2_, *y*_2_) on the borderline. As seen in Figure 6, a function detects the borderline between (*x*_1_, *y*_1_) and (*x*_2_, *y*_2_). Two squares with edge length 2*s *are marked by the dark color in a big square having a diagonal line from (*x*_1_, *y*_1_) to (*x*_2_, *y*_2_). We find the most possible border point in the two dark squares by using the same method in the first step. If this new border point is in the right-top square, we replace (*x*_2_, *y*_2_) by this point. Otherwise, we replace (*x*_1_, *y*_1_) by the new border point. Once (*x*_1_, *y*_1_) or (*x*_2_, *y*_2_) is updated, we continue to find the next border point in the same way. Repeat this procedure until the distance between the two points is less than *2s*. Connecting all points in such an orderly way, we obtain the borderline. It is noted that this method is convergent because the distance between two working points becomes smaller and smaller in this procedure.

3. We repeat step 2 until the borderline is closed. We thus obtain the whole closed borderline in the slice.

4. For a 3-D medical image, due to the similarity of neighboring slices, the proposed software can map the selected key border points of the slice onto the neighboring slice and use the method in step 1 to find the corresponding border points in the new slice. After that we adopt step 2 to detect the borderline between the border points. In this way, we are able to detect the borderlines in all the slices. From these borderlines we can finally construct the surface of the selected ROI.

Because the borderlines of other slices are detected on the basis of the first slice, selection of this slice greatly affects the quality of results. We suggest that this slice need contain the most representative information. If the change of two neighboring slices is large, we can optionally reselect the border point in the new slice instead of detecting the borderline by the computer. It is further noted that because the borderlines detected by the computer may be very irregular, we can use a cubic Bezier curve fitting technique to smooth the borderlines.

### FEM mesh Generation

Among several methods to automating mesh generation [9], the mesh with cuboidal elements is the fastest and most stable method to mesh an organ with irregular shape even though it may require more elements at the boundary. We therefore apply the cuboidal-element mesh to make this software applicable for complex cases. For instance, in Figure 3 the cloud-like parenchyma is dispersed in the fatty tissue. It is almost impossible to extract the exact geometry of parenchyma. In this case, most geometry-based methods are invalid. In the cuboidal mesh, elements are generated layer by layer and are automatically connected through the overlaid nodes. Given an element size, we can calculate how many slices each layer of elements spans. For simplicity, we assume the borderline of the central slice to be the borderline of that layer. Since the borderline consists of many points, we first build up a grid using the element size, move each border point to the closest cross-point of the grid, and remove the repeated points. We thus obtain the borderline denoted by the cross-points of the grid. It is noted the borderline may be entangled somewhere due to the numerical truncation. We need normalize the borderline so that it encloses a single-connected region and the distance between two neighboring point is equal to the size of the elements.

When we scan the single connected region, there exist two types of points on the borderline: jumping points and inertial points. If the left and right sides of a point are in the different states; i.e. one side is in the inside of the objective region and the other side is in the outside, then the point is called a jumping point. Otherwise, this point is called an inertial point. For instance, in an upstanding rectangle, all points on the left and right sides are jumping points, whereas the rest points on the top and bottom sides are inertial ones. On a closed borderline, each point is connected to two points. For a jumping point, the two neighboring points apparently have different values of *y *coordinate, whereas those for an inertial point do not. From this criterion we can identify the jumping points on the borderline. Once the points of the borderline are given, we can sort the points from top to bottom by *y *coordinate and from left to right by *x *coordinate. Then, for any *y *coordinate we can obtain a list of points with increasing *x *coordinates. During a horizontal scan for a fixed *y *coordinate, the number of jumping points in this list must be even, with which we obtain the pair-wise jumping points and find all the internal points between each pair of jumping points. Scanning the points from top to bottom, we can obtain all the internal points for the connected region. Then we can obtain the cuboidal-element mesh for this layer by mapping one point onto one element.

Because different elements may have different material properties, we need find the segmentation information for each element. From the grayscale histogram, we have defined the grayscale ranges corresponding to the tissues. Typically, an element may contain many voxels that belong to different tissues, whereas the FEM requires the element to be homogeneous. We count the number of voxels for each tissue in the element and assume the maximum one to be the material of the element. Thus, we can map the elements onto the different tissues as shown in Figure 5. We can thus mesh the object layer by layer and finally obtain the total FEM mesh, from which we can further calculate the volume of each tissue.

The surface information of the object is important for applying boundary conditions and mechanical loadings. This software uses the 2-D rectangular elements to describe the surface. Each 3-D cuboidal element has six rectangular faces. We collect the faces from all cuboidal elements. Thus, for an object containing *N *cuboidal elements, we can obtain 6*N *2-D rectangular elements. Obviously not all the rectangular elements are on the surface of the object. If an element is not on the surface, from the connectivity, another 2-D element containing the same nodes must exist which belongs to the neighboring cuboidal element. Eliminating each pair of these inside elements, we are able to obtain surface elements. We further input the mesh with segmentation information into an FEM program based on the required data format, assign material properties to tissues, and apply boundary conditions on the surface nodes. We can eventually calculate the mechanical deformation, internal stress and strain by the FEM software.

## Results and Discussion

### 3-D FEM Mesh and Material Properties

**Figure 7:**
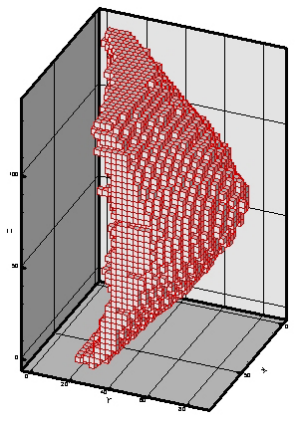
**3D FEM Mesh of the Breast. **The breast is meshed by cuboidal elements with a size of 2.8125 × 2.8125 × 3 *mm*. 14,902 elements and 18,486 nodes are generated. The breast is in the region as: 0 <*x *< 84.375 *mm*, 0 <*y *< 87.1875 *mm *and 0 <*z *< 135 *mm*.

To illustrate the capability of this software, we construct an FEM model of a woman breast and simulate the mechanical deformation with applied compressive forces. A set of CT image of the prone breast was acquired consisting of 512 × 512 × 243 voxels. The voxel size is 0.46875 × 0.46875 × 0.6 *mm*^3^. As an example, the 148^th ^slice is shown in Figure 1. The breast includes three kinds of tissues: fat, parenchyma, and tumor, which are represented by three grayscales as dark, gray, and light, respectively. Using the ImageParser package, we are able to mesh the breast by cuboidal elements with a size of 2.8125 × 2.8125 × 3 *mm*^3^. The breast is meshed into 14,902 elements with 18,486 nodes as shown in Figure 7. The tumor, parenchyma, and fatty tissue consist of 154, 5783, and 8965 elements, respectively. The surface of the breast includes 6,900 rectangular 2-D surface elements. The region of breast is defined as follows: 0 <*x *< 84.375 *mm*, 0 <*y *< 87.1875 *mm *and 0 <*z *< 135 *mm*. Here *x *is from left to right in a slice of the image, *y *is from the top to bottom, and *z *is from the first slice to the last slice. Corresponding to the human body, *y *represents the normal direction of the coronal plane, while *z *signifies the normal direction of the axial (transverse) plane (Figure 7).

Based on Krouskop et al. [13], the initial elastic moduli of three tissues are taken as 20 KPa for fat, 35 KPa for parenchyma, and 100 KPa for tumor. Because these tissues may undergo large (finite) deformation, we apply the Mooney-Rivlin nonlinear elastic (hyperelastic) model to describe the constitutive law for the finite deformation. Using the initial elastic moduli we can calculate the Mooney-Rivlin material constants as: *C*_01 _= 1,333 Pa, *C*_10 _= 2,000 Pa for fat; *C*_01 _= 2333.3 Pa, *C*_10 _= 3500 Pa for parenchyma; and *C*_01 _= 6,667 Pa, *C*_10 _= 10,000 Pa for tumor. It is noted that, due to the nonlinear characteristics, the elastic modulus for each tissue change as a function of deformation.

### FEM Modeling by ANSYS

ANSYS 7.0 [14] is the commercial nonlinear FEM software. We input the nodes and elements into ANSYS, and define the material models for three tissues. The applied compression with two flat-paddles is designed to simulate the clinical mammography examination. The ANSYS elastic contact model is adopted for the interaction between the breast tissue and the much more rigid paddle. The paddle's Young's modulus and Poisson's ratio are taken as 210 GPa and 0.3, respectively. During the compression process, the breast deforms. The contact area between the breast surface and the paddle increases automatically. The friction coefficient between the breast and the paddle is assumed to be 0.2. The boundary conditions are assumed that all nodes attached to thorax are constraint as *U*_*x *_= *U*_*y *_= 0, so that they can only move in the *z *direction for computational convenience. The two paddles move toward each other with a quasi-static strain rate. The maximum paddle movement is limited to be 13.5 *mm *(20% deformation) in the *z *direction.

### FEM Results

**Figure 8:**
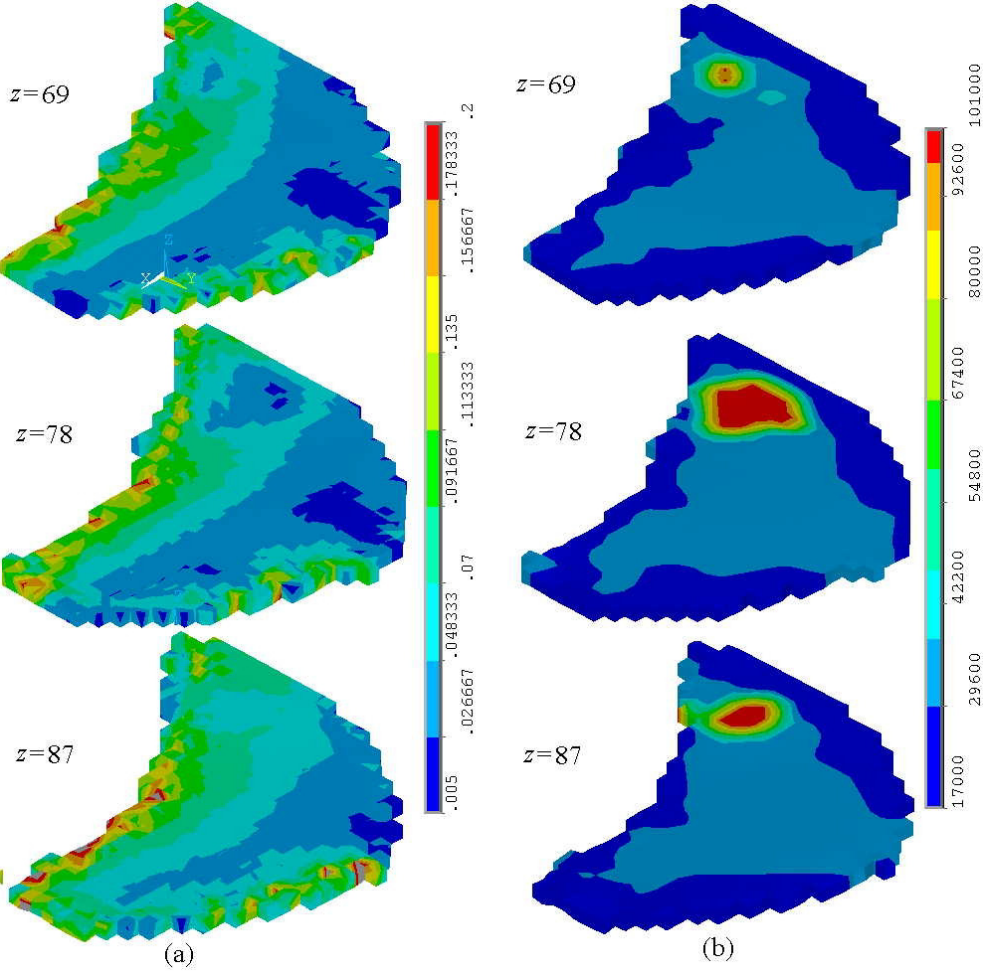
**The Strain and Tangent Young's Modulus Distribution. **The von Mises strain (a) and Tangent Young's modulus (b) distribution in the layers at *z *= 69, 78, 87 *mm *are illustrated. Because the tumor is much harder than other tissues, the Tangent Young's modulus is obviously higher and the strain is lower than those at the neighoring region.



To simulate the nonlinearly elastic deformation, we divide this compression process into 20 incremental steps. At each step the displacement, strain, and stress fields can be calculated. Figure 8 shows the von Mises strain and tangential Young's modulus distributions at the last step. The deformation-dependent tangential Young's modulus is defined as

where *σ*_*ε *_and *ε*_*e *_are the von Mises stress and strain, respectively. It is noted that, for linear elastic material, *E *is always a material constant. Because the material properties of skin, normal entity and tumor are all nonlinear, *E *should change during the process of compression. Figure 8(a) shows the von Mises strain for the sections at *z *= 69,78,87 *mm*. The strain around the thorax is quite significant due to the boundary constraint, whereas the strain close to skin is small because of the free boundary condition. In the region of tumor, it is shown that the strain is much smaller than that in the neighboring region because the tumor is much harder than other tissues. Figure 8(b) demonstrates that the tangential Young's modulus is no longer uniform even in the same tissue because its strain field is not uniform.

## Conclusions

The ImageParser system has been developed to create FEM mesh models from 3-D medical images. A semi-automatic method has been proposed to detect the ROIs from the context of complex image structures. The ROIs can be meshed into cuboidal elements and segmented based on the grayscale of the voxels. It has been demonstrated that, through a 3-D CT image volume of the woman breast, the ImageParser can effectively mesh the breast into cuboidal elements, and simulate the realistic nonlinear deformation responses of the breast tissues upon compression.

## Authors' contributions

LZS, GW, and MWV conceived and planned this research project. HMY and LZS designed and developed this software. TY prepared the CT image volume of the breast. TY, JW, and GW analyzed the CT images. HMY, LZS, and GW wrote the manuscript.
